# Analysis of root surface properties by fluorescence/Raman intensity ratio

**DOI:** 10.1007/s10103-017-2291-x

**Published:** 2017-07-25

**Authors:** Shino Nakamura, Masahiro Ando, Hiro-o Hamaguchi, Matsuo Yamamoto

**Affiliations:** Department of Periodontology, Showa University School of Dentistry, 2-1-1, Kitasenzoku, Ohta-ku, Tokyo, 145-8515 Japan

**Keywords:** Periodontal debridement, Periodontitis, Dental calculus, Raman spectroscopy, Diagnostic system, Hydroxyapatite

## Abstract

The aim of this study is to evaluate the existence of residual calculus on root surfaces by determining the fluorescence/Raman intensity ratio. Thirty-two extracted human teeth, partially covered with calculus on the root surface, were evaluated by using a portable Raman spectrophotometer, and a 785-nm, 100-mW laser was applied for fluorescence/Raman excitation. The collected spectra were normalized to the hydroxyapatite Raman band intensity at 960 cm^−1^. Raman spectra were recorded from the same point after changing the focal distance of the laser and the target radiating angle. In seven teeth, the condition of calculus, cementum, and dentin were evaluated. In 25 teeth, we determined the fluorescence/Raman intensity ratio following three strokes of debridement. Raman spectra collected from the dentin, cementum, and calculus were different. After normalization, spectra values were constant. The fluorescence/Raman intensity ratio of calculus region showed significant differences compared to the cementum and dentin (*p* < 0.05). The fluorescence/Raman intensity ratio decreased with calculus debridement. For this analysis, the delta value was defined as the difference between the values before and after three strokes, with the final 2 delta values close to zero, indicating a gradual asymptotic curve and the change in intensity ratio approximating that of individual constants. Fluorescence/Raman intensity ratio was effectively used to cancel the angle- and distance-dependent fluctuations of fluorescence collection efficiency during measurement. Changes in the fluorescence/Raman intensity ratio near zero suggested that cementum or dentin was exposed, and calculus removed.

## Introduction

Periodontitis is an infectious disease initiated in dental plaque by oral bacteria. Bacterial pathogenic factors, such as proteolytic enzymes, lipopolysaccharides, and fimbriae, disturb epithelial attachment and connective tissue structure, contributing to periodontal disease progression [1, 2]. Successful periodontal therapy is based on, in an ideal situation, complete removal of the virulent components of bacteria from root surfaces and results in the reattachment of epithelium, the fibroblast growing back, and connecting back to tooth cementum [3–5]. Scaling is a procedure which aims at the removal of plaque and calculus from the tooth surface. Root planning denotes a technique of instrumentation by which the softened cementum is removed and the root surface is made hard and smooth. Penetration of lipopolysaccharides to cementum is only within 10 μm [6, 7]. Treatment, including the elimination or the control of the biofilm infection and introduction of careful plaque control measures, in most, if not all, cases results in dental and periodontal health [8]. Thus, it does not mean there is no need to do over-instrumentation for healing periodontitis and regenerate periodontal tissue.

The presence of calcified deposition is typically evaluated using a periodontal probe with a walking stroke technique [9]. However, it is difficult to detect extremely small or burnished calculus deposits remaining on the root surface during initial preparation, because subgingival calculus is not directly visible. Recently, several different detection methods have been developed to identify whether calculus remains, of which the Perioscopy™ device (Perioscopy Inc., Oakland, CA, USA) is a minimally invasive diminutive endoscope that can be directly inserted into a periodontal pocket. Although visible observation is reliable for detection of residual calculus with that device, subjective judgment is an important factor, especially with inexperienced clinicians, and it has been reported that it is difficult to master the Perioscopy™ without long hours of training [10, 11]. Another available device is the Diagnodent™ Pen (KaVo, Biberach, Germany), an auto-fluorescence-based technology used to determine calculus by detecting fluorescence induced by laser radiation from a 655-nm diode. This device serves only as a fluorescence detector, so it is extremely influenced by microbial plaque or calculus. Calculus detection values vary among examiners because of inconsistent irradiation angles and distances to the root surface [12].

On the other hand, Raman spectroscopy, an optical technique that uses vibrational spectroscopy, is considered to be a promising tool for obtaining substance information. Without staining or pretreatment, Raman spectroscopy can provide qualitative and quantitative information regarding nearly all kinds of molecules via molecular fingerprints in the vibrational spectra. Furthermore, it is less affected by moisture and applicable for noninvasive real-time analysis. Because of its advantages, Raman spectroscopy is expected to have applications for various fields of medicine, such as cancer diagnosis [13], monitoring the effects of drugs [14], and rapid identification of pathogenic bacteria [15]. Furthermore, it has been applied to the pathology of hard tissues including bones and teeth, by obtaining Raman spectra of hydroxyapatite (HA) and organic components such as proteins [16–18]. It has also been reported that detailed Raman spectral analysis of HA was highly useful for assessments of decayed teeth [19, 20].

For effective utilization of Raman intensity as an internal standard for normalization of examiner-dependent fluorescence intensity, it is important to simultaneously detect fluorescence from calculus and Raman scattering from the tooth substrate from the same point receiving laser irradiation. The goal of the present study is that we will attempt to determine the existence of residual calculus on root surfaces by determining the fluorescence/Raman intensity ratio. Using a spectrometer with 785-nm excitation, simultaneous observations of fluorescence from calculus and Raman scattering from HA will be achieved. After canceling the angle- and distance-dependent fluctuations of fluorescence intensity, the fluorescence/Raman intensity ratio will provide accurate diagnostic information in regard to residual calculus.

## Materials and methods

### Samples

We examined 32 human teeth extracted during periodontal treatment and partially covered with dark-brown and bulky calculus on the root surfaces, after receiving informed consent from each patient. Plaque and blood on the root surfaces were removed with distilled water, then the teeth were dried and stored at room temperature until Raman measurements were performed. The study protocol was approved by the internal ethics committee of the Department of Periodontology of Showa University School of Dentistry (Tokyo, Japan).

### Removal of calculus and status of root surface

To investigate the influence of calculus removal on Raman measurement, the root surfaces of 32 extracted teeth were examined before and after calculus removal. A #5/6 curette (Gracey, Hu-Friedy, Leimen, Germany) was used to remove calculus, then the condition of each was evaluated based on the existence of calculus (roughness noted by investigator), following removal of calculus after scaling, root planning (SRP), and exposure of healthy cementum, and over-instrumentation, which indicated exposed dentin. The SRP of all of the root surfaces was performed by a dentist in the same way.

### Raman spectroscopy

All spectra were recorded using a portable Raman spectrophotometer (ProRaman-L, Enwave Optronics, Inc). The dental sample was placed onto a mechanical stage, then 785-nm, 100-mW laser irradiation was applied for fluorescence/Raman excitation. The laser beam was focused on the surface of the sample, with radius sizes of 0.5 and 2.0 mm used for the measurements. The exposure time for each measurement was 10 s, with spectra measured 10 times and then averaged.

### Normalization of fluorescence intensity

For normalization of fluorescence intensity, we used the Raman band intensity of HA. For the *ν*_1_ Raman band at ~960 cm^−1^, we used a least squares curve fitting method with Gaussian function to determine the PO_4_ symmetric stretch vibrational mode of HA, as follows

$$ \boldsymbol{f}\left(\boldsymbol{x}\right)={\mathbf{c}}_0+{\boldsymbol{c}}_1\boldsymbol{x}+\boldsymbol{A}{\boldsymbol{e}}^{-{\left(\raisebox{1ex}{$\left(\boldsymbol{x}-{\boldsymbol{x}}_0\right)$}\!\left/ \!\raisebox{-1ex}{$\boldsymbol{w}$}\right.\right)}^2} $$

Where the (**c**_0_ + ***c***_1_ ***x***) term indicates the linear offset of Gaussian function, and ***A***, ***x***_0_, and ***w*** indicate height, peak position, and band width, respectively, of Gaussian function. From the fitted result, the Raman band intensity of HA was estimated using the value of ***A*** and fluorescence intensity was estimated as **c**_0_ + ***c***_1_ ***x***_0_. Finally, by determining the ratio of these two values, the normalized fluorescence intensity (**c**_0_ + ***c***_1_ ***x***_0_)/**A** was calculated.

All data analysis procedures were performed using a commercially available software package (Igor Pro 6.3, Wave Metrics, Inc.).

### Histometry analysis

Seven of the extracted teeth selected randomly were measured along a single line every 0.5 mm from the coronal to apical side on an xy-stage. Prior to performing the measurements, small notches were set to indicate the beginning and end of the line. The results were documented with images and histological findings. Each embedded sample was cut into slices with a thickness of 1 mm and used for histometry analysis. Optical images were taken with a stereomicroscope (SD61, Olympus, Tokyo, Japan), a digital microscope, and scanning electron microscope (SEM) (VHX-D500, KEYENCE, Osaka, Japan). To validate whether cementum layer was recognized, tissue sections were stained with hematoxylin and eosin solution.

### Statistical analysis

Differences between each point were assessed by Wilcoxon rank sum test followed by Bonferroni multiple comparison test. A *p* value <0.05 was considered significant.

Statistical analysis was performed by SPSS 16.0 software (SPSS, Inc., Chicago, IL, USA).

## Results

### Changes in Raman spectrum based on dental root surface properties

**Fig. 1:**
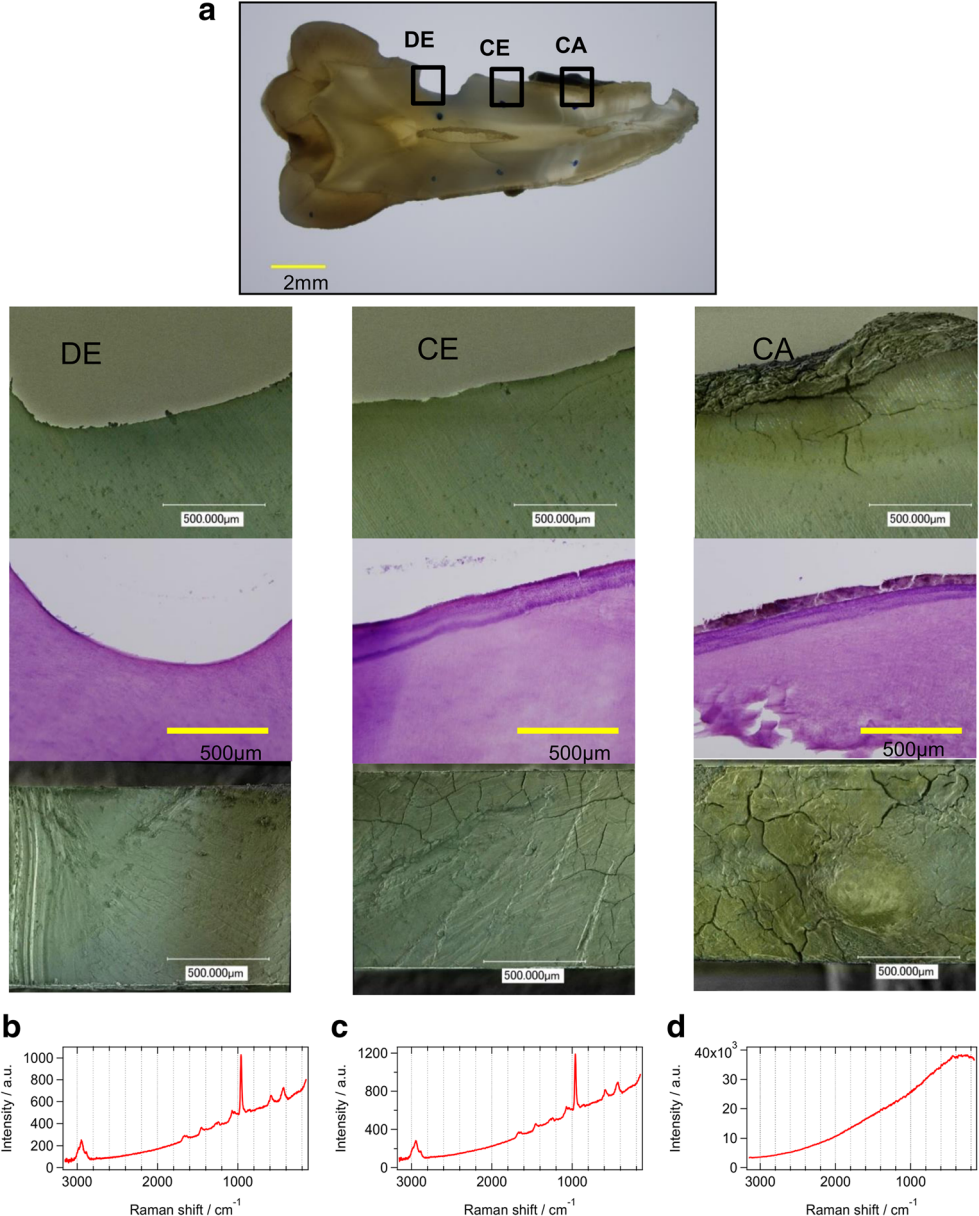
Changes in Raman spectrum based on dental root surface properties. Dentin (*DE*), cementum (*CE*), and calculus (*CA*) with a stereomicroscope (×40), a digital microscope with scanning electron microscope (×200), and H-E stain (×200) were confirmed (**a**). The raw Raman spectra of dentin, cementum, and calculus were recorded (**b**, **c**, **d**)

Typical Raman spectra were collected from the dentin, cementum, and calculus of each tooth, which were prepared by slicing into sections 1 mm thick. Each root surface point was observed histologically with a stereomicroscope, a digital microscope, and SEM. Based on histological analysis findings, three different surface structures of the root could be detected, which were confirmed to be dentin, cementum, and calculus (Fig. 1a).

Raman spectra were also recorded at the same points. With dentin and cementum, the level of fluorescence was low and a Raman band at 960 cm^−1^ was observed for HA. In addition, other HA Raman bands at 440 and 580 cm^−1^, as well as an organic compound Raman band at about 2940 cm^−1^ were observed (Fig. 1b, c). In contrast, almost no HA Raman band was observed from the calculus due to strong fluorescence intensity (Fig. 1d).

### Normalized Raman spectrum

**Fig. 2:**
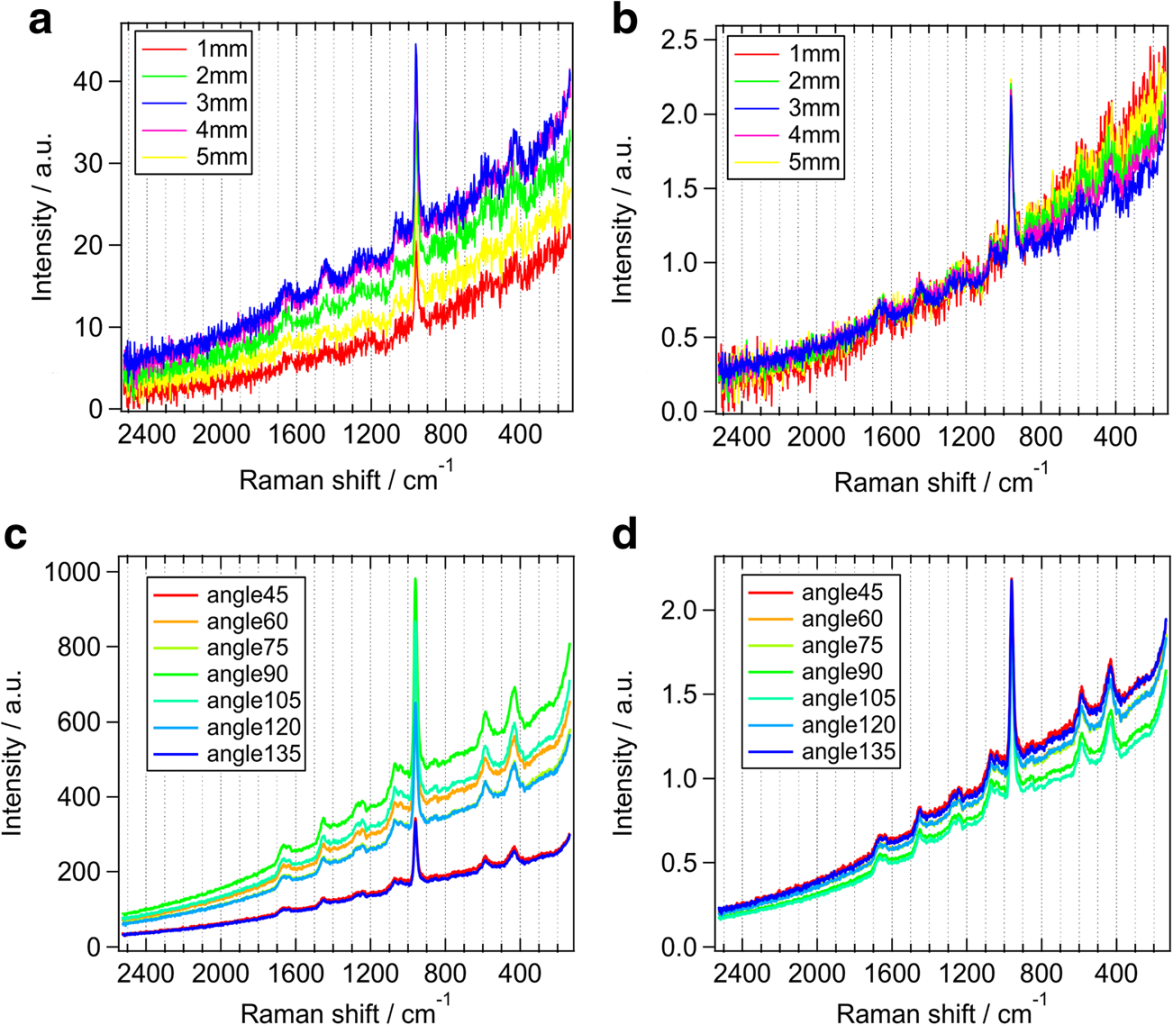
Normalized Raman spectrum. Raman spectrum were collected at the same point changing a focal distance from 5 to 1 mm of a laser beam (**a**) and a target radiating angle of it from 45° to 135° (**c**). Each spectrum were normalized at 960 cm^−1^ HA Raman band (**b**, **d**)

By canceling the angle- and distance-dependent fluctuations of collection efficiency, the fluorescence/Raman intensity ratio was calculated, namely normalization. Raman spectra were recorded from the same point after changing the focal distance of the laser from 5 to 1 mm and the target radiating angle from 45° to 135° (Fig. 2a, c). Each spectrum was normalized to the 960-cm^−1^ HA Raman band (Fig. 2b, d). After normalization, these spectra values were constant. After calculating the fluorescence/Raman intensity ratio, the standard deviations after normalization were smaller as compared to those obtained before that calculation (Table 1). Thus the intensity of fluorescence from minute calcium deposits could be quantified.

**Table 1:** Comparison fluorescence/Raman intensity ratios with before and after normalization

	Distance (mm)	Before	After	Angle (°)	Before	After
	5	13.83	1.23	45	174.43	1.11
	4	21.83	1.16	60	366.46	1.03
	3	22.81	1.10	75	319.85	1.02
	2	18.43	1.18	90	455.68	0.93
	1	10.63	1.20	105	391.12	0.88
				120	318.08	1.03
				135	166.06	1.08
Average (SD %)		16.80 (30.02)	1.09 (3.15)		313.10 (34.54)	1.01 (8.39)

### Changes in fluorescence intensity ratio corresponding to root surface properties

**Fig. 3:**
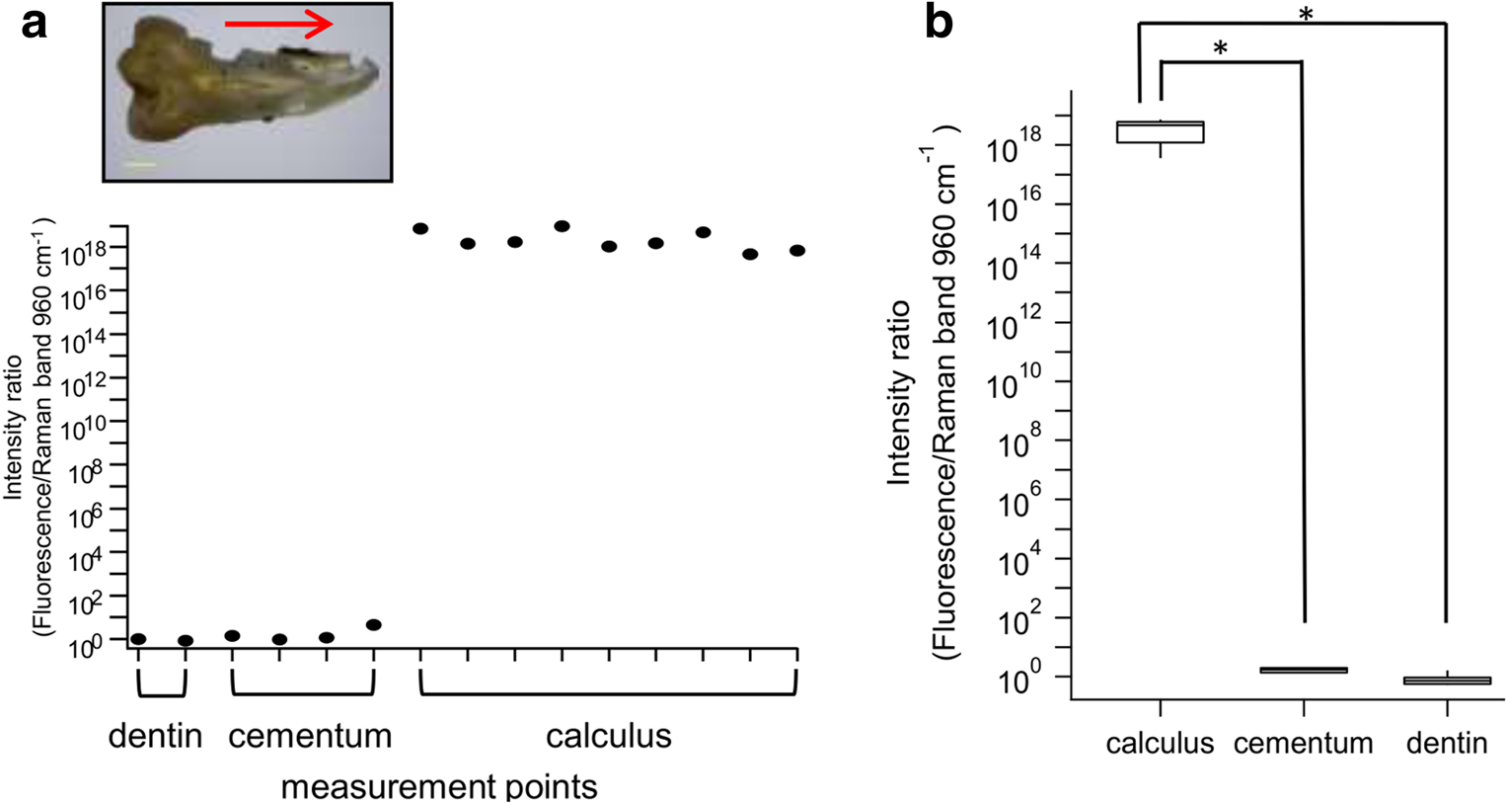
The change of fluorescence/Raman intensity ratio corresponds to root surface properties. The root surface of extracted tooth was scanned on a line in steps of 0.5 mm from coronal to apical. Typical changes of intensity ratio between dentin, cementum, and calculus (**a**). The normalized fluorescence intensity in calculus deposition region was much higher than that in cementum or dentin region. We found that the level of normalized fluorescence intensity in cementum was a little higher than that in dentin, but there was no significant difference (*p < 0.05, Wilcoxon rank sum test, *n* = 7) (**b**)

The root surface of each extracted tooth was scanned along a line in steps of 0.5 mm from the coronal to apical position. Normalized fluorescence intensity was calculated as described above, i.e., fluorescence/Raman intensity ratio, and then quantified. We found that the fluorescence/Raman intensity ratio of calculus deposit regions was much greater as compared to the cementum and dentin regions (Fig. 3a). In seven of the tested samples, the calculus region showed significant differences as compared to the cementum and dentin (Wilcoxon rank sum test followed by Bonferroni multiple comparison test). In addition, the level of normalized fluorescence intensity in cementum was slightly higher than that in dentin, though the difference was not significant (Fig. 3b). Calculus deposits are known to vary, thus the standard deviation for the intensity ratio from calculus had a wide range (Fig. 3b) and the change in intensity ratio according to change in calculus layer thickness was monitored.

### Change in fluorescence intensity ratio following three strokes of debridement

**Fig. 4:**
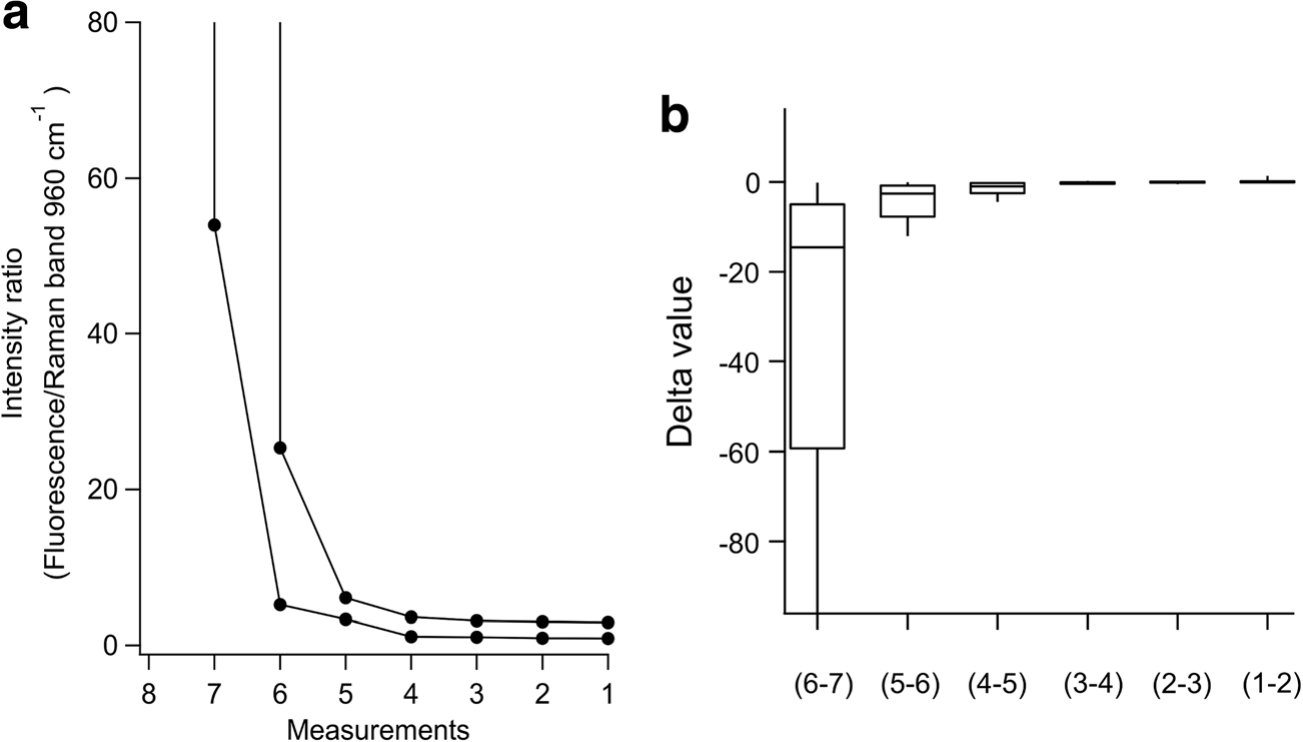
Change in fluorescence intensity ratio following three strokes of debridement. For all of the 25 extracted teeth, it was confirmed that fluorescence/Raman intensity ratio decreasing as dental calculus debridement. Typical changes of fluorescence/Raman intensity ratio in two teeth every three strokes were shown in **a**. Delta value was getting closer to zero gradually (*n* = 25) (**b**)

We also determined the fluorescence/Raman intensity ratio following debridement. Scaling of the root surface causes a decrease in the calculus layer [21]. In the present study, the root surfaces were scaled three times and then examined, and we repeated this several times. Figure 4a presents representative changes in fluorescence intensity ratio in association with calculus debridement. In 24 of the 25 teeth examined, the fluorescence/Raman intensity ratio decreased with calculus debridement.

For this analysis, the delta value was defined as the difference between the values before and after three strokes, with the final 2 delta values close to zero, indicating a gradual asymptotic curve (Fig. 4b) and the change in intensity ratio approximating that of individual constants. In addition, in the case of one tooth, the fluorescence/Raman intensity ratio showed a remarkable increase after calculus debridement even it decreased once (data not shown).

## Discussion

A previous study found that values for laser-induced fluorescence at an excitation wavelength of 655 nm on tooth root surfaces were strongly correlated with the presence of calculus [22], which we also found in the present study using a 785-nm excitation wavelength. The Diagnodent™ Pen (KaVo, Biberach, Germany) has been shown useful for obtaining accurate results in a clinical setting. However, it is difficult to distinguish whether the fluorescence intensity is the same as the fluorescence derived from calculus, by only measuring with the Diagnodent™ Pen. For accurate measurements, the laser should ideally be set to irradiate the sample surface at an appropriate distance and perpendicular angle, though in clinical settings those are difficult to maintain. Furthermore, quantitative values are difficult to obtain, because the collection efficiency of fluorescence is reduced without an appropriate laser irradiation angle and distance to the sample surface. Thus, the decrease of intensity does not mean that a signal from the healthy tooth surface is decreasing, and so we do not know whether the root surface is actually clean.

In the present study, Raman spectra from dentin or cementum showed that the level of fluorescence was much lower as compared to the calculus. Furthermore, specific HA Raman bands 960, 440, and 580 cm^−1^ were observed, which we considered to be evidence showing that the sound root surface was exposed. Also, since measurements of fluorescence intensity can vary because of irradiation angle or distance to the object, we focused on the 960-cm^−1^ HA Raman band and used normalization for performing an objective evaluation.

Hence, we used values showing the ratio of fluorescence intensity of calculus to the intensity of the HA Raman band, as previous studies reported that the amount of calculus present was in proportion to fluorescence intensity [22] and the HA Raman band provided quantitative information regarding minerals in biomaterials such as bones [16] and teeth [20]. In our study, the corrected spectrum values were normalized to the HA Raman band intensity at 960 cm^−1^ to produce quantitative results. Variations in measurement distance between the teeth and laser probe influenced the intensity of fluorescence (Fig. 2a), while normalization provided a constant value that was not influenced by fluctuations in distance (Table 1). Another factor that affects the intensity of fluorescence is radiation angle of the laser. According to the results obtained with samples irradiated from seven different directions, with the resulting values normalized in the same manner, the spectrum and the fluorescence/Raman intensity ratio were similar to our results shown in Fig. 2b. Thus, normalization removed the influence of the measurement conditions and analysis of the HA Raman band was useful to determine whether calculus was eliminated by debridement.

The fluorescence/Raman intensity ratio of calculus had an extremely high value in cases where the values for cementum and dentin were significantly lower (Fig. 3b). On the other hand, regardless of the presence of cementum, no significant differences were seen between cementum and dentin. Also some study reported that it was not necessarily to remove all cementum in periodontal therapy [23], thus we take this as the remaining issue to be investigated in future studies to make the difference between cementum and dentin clear. We used the 960-cm^−1^ HA Raman band for normalization because of its ability to fit, as that band is highly intense. However, that band also reflects tooth structure and differences in directionality of crystallization. Other HA Raman bands, such as the short wavelength region of 440 cm^−1^, should be examined for normalization of obtained values.

For the present experiments, we determined the fluorescence/Raman intensity ratio after every three scaling strokes and found that it decreased in accordance with calculus debridement (Fig. 4a). Diversity in HA crystal structure and mineral components has been shown, as biological HA-crystal formation is a complex process starting with nucleation of inorganic calcium phosphate and crystal growth is controlled by the organic extracellular matrix [24], while the final level of mineralization is influenced by the concentrations of calcium and phosphorus [25]. When those concentrations are low, foreign ions such as carbonate and fluoride become incorporated. In our study, the fluorescence/Raman intensity ratio based on HA Raman band intensity varied from 0.38 to 7.81. Thus, we could not determine a standardized value for the fluorescence/Raman intensity ratio of dentin or cementum. Since the fluorescence/Raman intensity ratio does not indicate root surface properties, we focused on the change in that ratio after debridement, which was defined as the delta value. Theoretically, the delta value after calculus removal is zero. For our study, we determined that scaling was accomplished when the delta value was close to zero, as shown in Fig. 4b. However, there was an exception when the fluorescence ratio increased even after calculus was removed. A previous study found that due to a reduced amount of mineral crystals and increased deposition of organic matrix, as well as organic by-products from bacterial activity, lesions present a lower mineral Raman signal and higher fluorescence background as compared to sound enamel [19]. We considered the possibility of decalcification in our exception case. In a future study, more detailed analysis is necessary to distinguish between cementum and dentin, which may be possible by analyzing the Raman band at 440 cm^−1^, because of the lower sensitivity of the crystal structure.

The limitations of Raman Spectroscopy are long acquisition times and the need to use high laser power. When the intensity of calculus auto-fluorescence is weaken or the calculus is almost removed, it is difficult to decide whether the fluorescence is weakened due to the way the laser is applied or calculus is really removed. Even though it is time consuming to obtain spectra when calculus auto-fluorescence is weak, it is still useful to use Raman spectroscopy in such a situation.

In summary, we found that changes in the fluorescence/Raman intensity ratio indicated alteration of root surface properties. A value near zero showed that cementum or dentin was exposed, and calculus removed. Our findings are expected to be incorporated as part of an objective method for evaluation of root debridement.

All procedures performed in studies involving human participants were in accordance with the ethical standards of the institutional and/or national research committee and with the 1964 Helsinki declaration and its later amendments or comparable ethical standards.
